# Venous thromboembolism in transgender women

**DOI:** 10.1590/1677-5449.202201201

**Published:** 2023-01-13

**Authors:** Marcos Arêas Marques, Marcelo Melzer Teruchkin, André Luiz Malavasi Longo de Oliveira

**Affiliations:** 1 Universidade Federal do Estado do Rio de Janeiro - UNIRIO, Rio de Janeiro, RJ, Brasil.; 2 Universidade do Estado do Rio de Janeiro - UERJ, Rio de Janeiro, RJ, Brasil.; 3 Universidade Federal do Rio Grande do Sul - UFRGS, Porto Alegre, RS, Brasil.; 4 Universidade de São Paulo - USP, Faculdade de Medicina - FM, São Paulo, SP, Brasil.

The terms transgender and gender-nonconformity describe a situation in which a person’s gender identity differs from the external sexual anatomy they were born with. The objectives of gender-affirmation in transgender women are to suppress male characteristics and induce female characteristics, to the extent possible. Gender-affirmation can encompass hormone therapy (HT), via a variety of routes, and affirmation surgery, in addition to other procedures, such as depilation and speech therapy.^1^^,^^2^

Compared with cisgender individuals, transgender women have greater prevalence of anxiety, depression, use of illicit substances and tobacco, and sexually transmitted diseases, such as human immunodeficiency virus infections, and it is estimated that more than 40% attempt suicide at some point in life.3 These data can be explained by exposure to stress factors, such as stigmatization (discrimination, rejection, and victimization) and identity concealment.^3^

Epidemiological data suggest that 0.3 to 0.6% of the adult population are transgender (around 25,000,000 transgender people worldwide),^4^^-^8 but the true prevalence is dependent on the definition used to classify the population. For example, studies that only include people who have had HT or gender affirmation surgery have reported prevalence of 7 to 9 per 100,000 people.7 However, in studies that have included transgender status based on self-report, prevalence was approximately 871 per 100,000 people.^7^^-^9 Provision of physician-guided gender affirmation HT has shown improved quality of life and reduces the disorders observed in this population.^3^

## THE OBJECTIVES OF HT

The usual objective of HT in a transgender woman is to induce physical changes that are aligned with her gender identity,^10^ maintaining hormone levels within the normal physiological range for the target sex. This includes suppression of endogenous hormones of the original sex and substituting them with hormones consistent with the stated sexual gender.

Estrogens used for HT in transgender women can basically be divided into two categories: natural human hormones (17β-estradiol [E2], estrone [E1] and estriol [E3]) and non-human derivatives, which include derivatives of pregnant mare’s urine (conjugated equine estrogens [CEE]) and esterified estrogens of vegetable origin.^11^ The dose used is normally higher than is employed for hormone replacement therapy (HRT) in cisgender women and depends on the physical changes targeted, the type of estrogen used, and the route of administration.^12^

Several different studies have demonstrated an increased risk of venous thromboembolism (VTE) in transgender women who are on HT, which is related to the type and dosage of the hormones employed and, primarily, to route of administration.^11^ However, the majority of data are extrapolated from clinical studies investigating contraception and HRT.^11^

Oral administration induces the hepatic first-pass effect, with increased pro-thrombotic factors, whereas non-oral routes, and transdermal administration in particular, do not appear to induce increased VTE.^11^ This can be a determinant factor in choice of HT, making transdermal administration the preferred route in transgender women with a personal or family history of VTE or those who have thrombophilia.

It should be emphasized that HT is not an elective treatment in this population, but an absolute necessity to achieve the desired phenotype. In many places, these women are at the margins of society and cannot access professionals who are able to prescribe HT. As a consequence, estrogens are very often obtained illegally and taken on the person’s own initiative, without professional guidance on the safest composition, dosage, and route of administration. Another point to be considered is that non-oral HT presentations are normally more expensive than oral preparations and thus inaccessible to the majority of people. One feasible strategy to attenuate the risk of VTE in groups at risk is to initiate prophylactic anticoagulation simultaneously with HT, especially for the first 6 to 12 months of treatment.^12^

Several studies have shown that the CEE most used in the United States are more thrombogenic than E2, which is the type most used in Europe.^11^ A retrospective study with more than 1,000 participants estimated that incidence of VTE ranged from 2 to 6% in transgender women treated with oral ethinylestradiol,^13^^,^^14^ which was approximately 20 times greater than the rate in the cisgender male control population. In a follow-up study with the same cohort, no increase in VTE risk was observed in users of estrogen preparations, except for those on ethinylestradiol.^4^^,^^15^ In 214 transgender women using oral or transdermal estradiol or estradiol gel, VTE was observed in 11 cases (5.1%).^16^ No events were observed in control groups of cisgender men or women.^16^

Another cohort study based on electronic patient records analyzed 2,842 transgender women paired to approximately 48,000 cisgender men and 48,000 cisgender women, showing that the transgender women had higher VTE incidence than both control groups.^17^ The majority of these transgender women were taking oral estradiol at a mean daily dose of 4 mg, which was the same dose as that administered to those who did not have VTE. The difference became more pronounced during follow-up of these patients, observing an increased absolute risk at 2 and 8 years of 4.1 and 16.7, respectively, per 1,000 people, compared to cisgender men and of 3.4 and 13.7, respectively, per 1,000 people, in relation cisgender women. This pattern is different from what is observed in postmenopausal women on HRT, among whom the risk of VTE is greatest in the first year and falls progressively over time. These data suggest that long term monitoring is essential in this population.

Although data are not available on the risk of VTE in transgender women on HT who undergo surgery, consideration should be given to suspending HT from 2 to 4 weeks before major surgery involving immobilization. Since this hiatus is undesirable for the majority of patients, an alternative option is to maintain HT and add one point to the patient’s Caprini score, prescribing thromboprophylaxis in accordance with the guidelines. Once such individuals have completely recovered and have returned to normal activities, HT with estrogen can be restarted, which is normally within 4 weeks.^18^ The prevalence of thrombophilias appears not to differ between the transgender population and the general population. Routine screening prior to HT is therefore not suggested.^19^

## ANDROGEN SUPPRESSION THERAPY

The majority of gender affirmation HT regimens for transgender women also include a second drug, used with the objective of suppressing production or countering the effects of androgens, particularly testosterone.11 The drug most often used for this is sprinolactone, a potassium-sparing diuretic that interacts with steroid hormone receptors, especially androgen receptors, inhibiting production of androgens and of 5α-reductase, an enzyme that coverts testosterone into dihydrotestosterone.^11^ Other drugs that can be used for this purpose include 5α-reductase inhibitors (finasteride), androgen receptor blockers (flutamide), progestins, and gonadotropin-releasing hormone agonists. There are no associations between these medications and increased incidence of VTE.

Finally, is it is important to emphasize that the transgender population exhibits peculiarities inherent to use of hormone therapy at supraphysiological dosages, difficulties with access to specialist medical services, and use of medications that are inappropriate for human use, which, in the final analysis, expose this vulnerable population to higher incidences of underdiagnosed and under-reported complications. Occurrence of VTE in the transgender population is one of the many facets that modern medicine must deal with.
