# Supplementary material for: Venous thromboembolism in transgender women
Source: J Vasc Bras. 2023 Jan 13;21:e20220120. doi: 10.1590/1677-5449.202201201 (PMC9844815; doi:10.1590/1677-5449.202201201)
Supplement: Translation

# Translation: Tromboembolismo venoso em mulheres transgênero

**DOI:** 10.1590/1677-5449.202201202

**Authors:** Marcos Arêas Marques, Marcelo Melzer Teruchkin, André Luiz Malavasi Longo de Oliveira

**Affiliations:** 1 Universidade Federal do Estado do Rio de Janeiro - UNIRIO, Rio de Janeiro, RJ, Brasil.; 2 Universidade do Estado do Rio de Janeiro - UERJ, Rio de Janeiro, RJ, Brasil.; 3 Universidade Federal do Rio Grande do Sul - UFRGS, Porto Alegre, RS, Brasil.; 4 Universidade de São Paulo - USP, Faculdade de Medicina - FM, São Paulo, SP, Brasil.

Os termos transgênero e incongruência de gênero descrevem uma situação em que a identidade de gênero de um indivíduo difere da anatomia sexual externa no nascimento. Os objetivos da afirmação de gênero na mulher transgênero visam à supressão, dentro do possível, das características masculinas e à indução de características femininas. A afirmação de gênero pode incluir terapia hormonal (TH), por diversas vias, e cirurgias de afirmação, além de outros procedimentos, como depilação e fonoaudiologia^1^^,^^2^.

Em comparação a indivíduos cisgênero (ou seja, não transgênero), as mulheres transgênero têm maior prevalência de ansiedade, depressão, uso de substâncias ilícitas e tabaco e doenças sexualmente transmissíveis, como infecção pelo vírus da imunodeficiência humana, e estima-se que mais de 40% tentaram suicídio em algum momento^3^. Esses dados podem ser explicados pela exposição a fatores de estresse, como a estigmatização (discriminação, rejeição e vitimização) e ocultação de identidade^3^.

Dados epidemiológicos sugerem que de 0,3 a 0,6% da população adulta é transgênero (cerca de 25.000.000 pessoas transgênero em todo o mundo)^4^^-^8, mas a real prevalência depende da definição usada na classificação dessa população. Por exemplo, em estudos que incluem apenas indivíduos que passaram por TH ou cirurgia de afirmação de gênero, a prevalência relatada foi de 7 a 9 por 100.000 pessoas^7^. No entanto, em estudos que incluem o *status* de transgênero com base em autorrelato, a prevalência foi de aproximadamente 871 por 100.000 pessoas^7^^-^9. O fornecimento de TH de afirmação de gênero orientada por médicos demonstrou melhorar a qualidade de vida e reduzir os transtornos citados nessa população^3^.

## OBJETIVOS DA TH

O objetivo usual da TH na mulher transgênero é induzir mudanças físicas que combinem com a identidade de gênero^10^, mantendo os níveis hormonais na faixa fisiológica normal para o sexo-alvo. Isso inclui suprimir hormônios endógenos da identidade de gênero original e substitui-los com hormônios consistentes com o gênero sexual afirmado.

Os estrógenos utilizados na TH para mulheres transgênero basicamente se dividem em duas categorias: os humanos naturais (17β-estradiol [E2], estrona [E1] e estriol [E3]) e os derivados não humanos, que incluem derivados de urina de éguas grávidas (conjugados estrógenos equinos [CEE]) ou esterificados de fontes vegetais^11^. A dose utilizada, normalmente maior que a usada para terapia de reposição hormonal (TRH) na mulher cisgênero, depende das mudanças físicas desejadas, do tipo de estrógeno e da via de administração^12^.

Vários estudos demonstraram um risco aumentado de tromboembolismo venoso (TEV) nas mulheres transgênero em uso de TH, o qual está relacionado ao tipo e às doses utilizados e, principalmente, à via de administração^11^. Porém, a maioria dos dados é extrapolada de estudos clínicos avaliando contracepção e TRH^11^.

A administração por via oral induz o efeito de primeira passagem hepática, com aumento dos fatores pró-trombóticos, enquanto as vias não orais, particularmente a via transdérmica, não parece induzir aumento de TEV^11^. Isso pode ser determinante na escolha da TH, em que a via transdérmica passa a ser a via preferencial em mulheres transgênero com história pessoal ou familiar de TEV e/ou portadoras de trombofilias.

Vale ressaltar que, para essa população, a TH não é eletiva, mas sim uma necessidade absoluta para se obter o fenótipo desejado. Em muitos lugares, essas mulheres estão à margem da sociedade e não possuem acesso a profissionais habilitados para prescrevê-la. Como consequência, muitas vezes os estrógenos são obtidos de forma ilegal e ingeridos por conta própria, sem orientação profissional quanto à composição, às doses e às vias seguras. Outro ponto para reflexão é que as vias não orais da TH são usualmente mais caras do que a via oral e, portanto, inacessíveis para a maioria. Uma estratégia plausível para atenuar o risco de TEV em grupos de risco é o início concomitante da anticoagulação profilática com a TH, especialmente nos primeiros 6 a 12 meses de tratamento^12^.

Diversos estudos mostraram que os CEE mais utilizados no Estados Unidos são mais trombogênicos do que o E2, que é o mais utilizado na Europa^11^. Em um estudo retrospectivo com mais de 1.000 participantes, a incidência de TEV foi de 2 a 6% em mulheres transgênero tratadas com etinilestradiol oral^13^^,^^14^, uma taxa aproximadamente 20 vezes maior do que a da população masculina cisgênero controle. Em um estudo de acompanhamento da mesma coorte, nenhum risco aumentado de TEV foi observado em usuários de preparações de estrógenos, exceto etinilestradiol^4^^,^^15^. Entre 214 mulheres transgênero que receberam estradiol por via oral, transdérmica ou gel de estradiol, foi observado TEV em 11 casos (5,1%)^16^. Nenhum evento foi observado nos grupos de controle de homens ou mulheres cisgênero^16^.

Um outro estudo de coorte baseado em prontuários eletrônicos incluiu 2.842 mulheres transgênero pareadas com aproximadamente 48.000 homens e 48.000 mulheres cisgênero e mostrou que as mulheres transgênero tiveram uma incidência maior de TEV do que ambos os controles^17^. A maioria das mulheres transgênero estava recebendo estradiol por via oral em dose diária média de 4 mg, a mesma administrada para aqueles que não tiveram TEV. A diferença foi mais pronunciada no seguimento desses pacientes, em que houve aumento do risco absoluto entre 2 e 8 anos de 4,1 e 16,7, respectivamente, por 1.000 pessoas em relação aos homens cisgênero e de 3,4 e 13,7, respectivamente, por 1.000 pessoas em relação às mulheres cisgênero. Esse padrão foi diferente daquele observado em mulheres na pós-menopausa usuárias de TRH, nas quais o risco de TEV mostrou-se maior no primeiro ano de uso, diminuindo progressivamente com o tempo. Esses dados sugerem que o monitoramento a longo prazo seria fundamental nessa população.

Embora os dados sobre o risco de TEV em mulheres transgênero em TH submetidas a cirurgia não estejam disponíveis, deve-se considerar interromper a TH de 2 a 4 semanas antes de cirurgias de grande porte com imobilização. Como essa pausa é indesejada para a maioria dos pacientes, uma alternativa é manter a TH e adicionar um ponto a mais no escore de Caprini, realizando a tromboprofilaxia de acordo com as diretrizes. Uma vez que esses indivíduos estejam totalmente recuperados e tenham retornado às suas atividades usuais, a TH com estrógeno pode ser retomada, normalmente, dentro de 4 semanas^18^. A prevalência de trombofilias parece ser a mesma na população transgênero e na população em geral. Portanto, a sua triagem de rotina pré-TH não é sugerida^19^.

## TERAPIA DE SUPRESSÃO ANDROGÊNICA

A maioria dos regimes de TH para afirmação de gênero em mulheres transgênero inclui uma segunda droga, com o intuito de suprimir a produção ou contrapor os efeitos dos androgênios, particularmente da testosterona^11^. A droga mais utilizada para esse objetivo é a espironolactona, um diurético poupador de potássio que possui interação com receptores de esteroides hormonais, especialmente os receptores de androgênio, inibindo a produção de androgênios e da 5α-redutase, uma enzima conversora de testosterona em di-hidrotestosterona^11^. Outras drogas utilizadas para esse fim são os inibidores da 5α-redutase (finasterida), os bloqueadores dos receptores androgênicos (flutamida), as progestinas e os hormônios agonistas/antagonistas da secreção de gonadotropina. Não há associação clara entre esses medicamentos e aumento da incidência de TEV.

Por fim, é importante salientar que a população transgênero apresenta peculiaridades inerentes ao uso de TH em doses suprafisiológicas, dificuldade de acesso a serviços médicos especializados e uso de medicações inadequadas ao ser humano, o que, em última análise, expõe essa população vulnerável a maiores incidências de complicações subdiagnosticadas e subnotificadas. A ocorrência de TEV na população transgênero é uma das muitas vertentes com a qual a medicina moderna precisará se preocupar.
